# Transcutaneous vagal nerve stimulation for the treatment of trauma- and stressor-related disorders: systematic review of randomised controlled studies

**DOI:** 10.1192/bjo.2025.10057

**Published:** 2025-08-01

**Authors:** Tarek Benzouak, Chad Danyluck, Sasha Gunpat, Steve E. Amougou, Rami A. Hamoudeh, Michael A. Prudencio-Brunello, Jude Nachabe, Febin Edwin, Steve Kisely, Sanjay Rao

**Affiliations:** 1 Faculty of Medicine, McGill University, Quebec, Canada; 2 Department of Psychiatry, University of Ottawa, Ontario, Canada; 3 Department of Psychology, Carleton University, Ontario, Canada; 4 Department of Psychology, Concordia University, Quebec, Canada; 5 Departments of Psychiatry, Community Health & Epidemiology, Dalhousie University, Nova Scotia, Canada; 6 Department of Psychiatry, University of Queensland, Queensland, Australia

**Keywords:** Trauma and stressor related disorders, post traumatic stress disorder, vagal nerve stimulation, systematic review

## Abstract

**Background:**

Vagal nerve stimulation (VNS) has recently emerged as a prospective therapeutic approach for addressing trauma- and stressor-related disorders (TSRDs).

**Aims:**

We assessed findings from randomised controlled trials for the safety and efficacy of VNS as a viable treatment for TSRDs.

**Method:**

We systematically searched Medline, Embase, PsycINFO, CINAHL, Web of Science, Cochrane Central databases, trial registries, preprint servers and Google Scholar from inception to December 2023. Rayyan software was used for screening procedures. Two reviewers independently completed data extraction based on the inclusion criteria.

**Results:**

We synthesised data by using a narrative approach. A total of 322 abstracts were identified and assessed, and seven studies were included in the review. Based on evidence synthesis, the present state of VNS as a treatment intervention for TSRDs, namely post-traumatic stress disorder (PTSD), is limited and does not meet clinical expectations. The overall certainty of evidence was very low. However, evidence shows that VNS may alter and reduce specific aspects associated with PTSD phenomenology, including the reduction of anger responses and the attenuation of hyperarousal during psychological interventions.

**Conclusions:**

Although preliminary analyses provide evidence that transcutaneous VNS temporarily increases parasympathetic activity under specific conditions, these effects appear to be short-lasting, and the impact of repeated administration on long-term autonomic function remains unknown. Future randomised control trials should evaluate the therapeutic efficacy of VNS for treating TSRDs.

Trauma- and stressor-related disorders (TSRDs) share a common aetiological pathway and are characterised by exposure to a traumatic or severely stressful event that precipitates illness.^1^ The pathogenesis of TSRDs is multifaceted, involving a complex interplay of neurobiological, psychological, behavioural, endocrine and immunogenic disruptions. The primary treatment for TSRDs is psychotherapy, including cognitive–behavioural therapy, cognitive processing therapy, prolonged exposure therapy and eye movement desensitisation and reprocessing.^2^ Although many individuals who undergo psychotherapy treatments show improvements, drop-out rates are high, and many still maintain their diagnosis following treatment.^3^ Physical interventions, such as deep brain^4,5^ and transcranial magnetic^6,7^ stimulation, have also shown promise for the treatment of TRSDs.^8^ However, it remains unclear whether attrition is as problematic as in psychotherapy. Vagal nerve stimulation (VNS) has recently emerged as an additional potential therapeutic strategy for the treatment of TSRDs.^9,10^

First approved for the treatment of seizures in palliative care,^11^ the use of VNS offers therapeutic relief from various illnesses, with meta-analytic evidence for its efficacy for the treatment of migraine-related pain,^12^ drug-resistant epilepsy^13^ and treatment-resistant depression.^14^ Modalities of VNS have traditionally been invasive, requiring the implantation of an electrode around the left vagus nerve and a combined battery and pulse generator below the clavicle. Although placement required general anaesthesia, it was considered a risky surgical procedure given the location of the electrode near the carotid artery.^15^ Recent technological advances have enabled the development of transcutaneous VNS (tcVNS), which delivers targeted electrical impulses via a non-invasive electrode array positioned at the cervical or auricular branch of the vagus nerve.^16,17^ This approach circumvents the surgical risks associated with traditional VNS while maintaining therapeutic neuromodulatory capabilities through precisely calibrated electrical parameters. The portable nature of tcVNS devices permits patient-administered treatment in ambulatory settings, representing a significant advancement in therapeutic neuromodulation accessibility.^18^

Most research on the development and persistence of TSRDs has focused on post-traumatic stress disorder (PTSD). Studies of the pathophysiology and neuroimmune-endocrine axis have chiefly been completed on PTSD, and have provided insight into the complex impact these disorders have on the autonomic nervous system (ANS) and its influence on circulating levels of interleukin-6, interleukin-1β, tumour necrosis factor-α, interferon-γ,^19^ cortisol^20,21^ and cardiovascular signalling molecules such as norepinephrine.^22^ Studies have reported associations between ANS abnormalities and evidence of decreased heart rate variability (HRV) compared with controls, as well as general ANS dysfunction.^23,24^ Specifically, abnormalities in high-frequency HRV components have been observed in individuals with PTSD, indicating compromised parasympathetic nervous system (PNS) function, which plays a critical role in stress response and emotional regulation. Considering the role of the vagus nerve in orchestrating parasympathetic activity, tcVNS could therefore represent a viable option for people with trauma- and stress-induced psychiatric illnesses.

The involvement of the PNS and sympathetic nervous system (SNS) branches of the ANS within the induction (i.e. the initial physiological response to trauma) and prognosis of PTSD has been well-characterised within the literature.^25–27^ Moreover, functional deficits common to PTSD – altered arousal, reactivity, intrusive thoughts, negative changes in mood and cognition, and extreme social withdrawal – are linked to physiological experiences, such as autonomic arousal.^28^ Furthermore, context has been suggested to play a role; for instance, changes in physiological response (e.g. skin conductance, heart rate) are known to increase in magnitude in the presence of perceived threat, with people with PTSD showing higher physiological responses than healthy controls.^21,29,30^ Indeed, both SNS and PNS systems are known to modulate stress^31^ and trauma responses, and alterations in their activity within the context of PTSD result in greater symptom severity.^32^ In addition to changes in ANS functioning, disruptions in the negative feedback of the hypothalamic-pituitary-adrenal (HPA) axis are a consistent abnormality observed in individuals with PTSD.^33^ During the heightened stress response, increased concentrations of norepinephrine and epinephrine lead to the release of cortisol, initially amplifying the SNS response and then diminishing it by negative feedback mechanisms.^32^ Altered profiles of inflammatory biomarkers in individuals with PTSD are further robustly linked to ANS alterations,^34,35^ with pro-inflammatory markers demonstrated to be linked to PTSD severity.^36^ In patients with PTSD, diminished PNS activity – crucial for modulating heart rate under quiescent states and exerting a tonic inhibitory effect on cardiac function – indicates an impaired ability for physiological regulation and recovery.^37,38^ This PNS dysregulation underscores an autonomic imbalance that contributes to the pathophysiology of PTSD. tcVNS, by directly enhancing parasympathetic output, presents a novel and targeted approach to rectify this imbalance. The therapeutic promise of tcVNS, predicated on its capacity to restore parasympathetic function, warrants further investigation as a potential intervention for the autonomic dysregulation characteristic of PTSD.

Notably, two theories provide a framework for the potential benefits of tcVNS. Founded on assumptions of phylogenetic heritage,^39^ the polyvagal theory posits that PNS activity is susceptible to cues of threats, with its activity maintained by perceptions of safety.^40^ Within this theoretical framework, experiences that promote vagal tone result in improved PTSD symptom experiences. Similarly, the neurovisceral integration theory (NVIT) posits that cardiac vagal tone reflects emotional and cognitive experiences, influencing stimuli processing and attentional processes.^41,42^ According to NVIT, imbalances in the integration of autonomic responses with central neural activities may contribute to the cognitive and emotional regulation challenges observed in TSRDs such as PTSD, providing a theoretical basis for understanding these dysfunctions.^43^ Notably, both theories converge on the premise that the activity of the vagal nerve is decreased by traumatic exposure, and that increases in vagal and cardiac modulation result in improved cognitive and emotional outcomes. Recent meta-analysis further provides some degree of support for these theories, demonstrating a small but present association between PTSD symptom severity and respiratory sinus arrhythmia (RSA), a known measure of vagal activity.^23^ These theories both provide a neurophysiological basis for the interaction between traumatic stress-related physiological changes and the development of TSRDs.^40^ However, these theories remain tentative with uncertain trial-based evidence for treating people with TSRDs. As both polyvagal theory and NVIT heavily emphasise the vagus nerve’s role in managing stress and trauma,^26,43^ evaluating tcVNS within the context of TSRDs offers a compelling opportunity to test these theories among psychiatric populations. This approach is grounded in the premise that tcVNS’s direct modulation of vagus nerve activity can provide insights into the nerve’s contribution to stress and trauma resilience.

The current systematic review synthesises evidence on trial literature associated with tcVNS interventions for TRSDs, evaluating the effect of tcVNS on psychiatric symptoms, psychological experiences, autonomic and central nervous system response and immunological changes. The aim was to characterise the current state of evidence and inform researchers, physicians and policy makers involved in this method of care. Beyond evaluating efficacy, the findings were further used to evaluate the current applicability of both polyvagal theory and NVIT within the context of PTSD treatment by vagal stimulation.

## Method

This systematic review was conducted in accordance with the PRISMA guidelines^44^ and, where possible, the recommendations of the synthesis without meta-analysis in systematic reviews (SWiM).^45^ The PRISMA checklist is available in the Supplementary Material, available at https://doi.org/10.1192/bjo.2025.10057. The SWiM approach included estimations of common effect sizes where applicable, and assessments of study quality and evidence credibility. The protocol was preregistered on Open Science Framework (https://osf.io/qm4jr) on 17 March 2022, and PROSPERO, an international database of prospectively registered systematic review protocols, on 14 April 2022 (CRD: 42022318838).

### Search strategy

Medline, Embase, PsycINFO, CINAHL, Web of Science and Cochrane Central databases, trial registries (i.e. ISRCTN, ClinicalTrials.gov and the National Institutes of Health clinical trial registry), preprint servers (i.e. MedRxiv and PsyArXiv) and Google Scholar were searched from inception to 23 December 2023. References from review articles, opinion publications and listed publications associated with trial registries were hand-searched and screened.

A combination of MeSH terms and keywords associated with VNS and TSRDs were used to identify VNS trials inclusive of people living with TSRDs. We imposed no language, setting or study design restrictions during the search. The search strategy was synthesised by one of the authors (T.B.) and was reviewed independently by three senior authors (C.D., S.R. and S.K.). The search strings used for each database are available within Supplementary File 2.

Rayyan software^46^ was used for screening procedures and the tracking of reasons for exclusion. Two independent reviewers (T.B. and S.G.) conducted title, abstracts and full-text screening, and any disagreements were resolved by a third author (C.D.). All included studies were further cross-validated for inclusion by a senior author (S.R.). The search was further updated by (S.E.A. and R.A.H.), and similarly, conflicts were resolved by a third independent reviewer (T.B). The initial screening was completed in April 2022, updated in March 2023 and once again on 23 December 2023, with all updates utilising the same search strategy.

### Selection criteria

Randomised controlled studies met inclusion criteria if they (a) administered a VNS-based intervention; (b) included adult patients (i.e. ≥18 years of age) diagnosed with TSRD, as defined and operationalised by a diagnostic manual (e.g. the DSM or ICD) and (c) used clinically validated outcomes measures for TSRD and a quantitative assessment of VNS-related response within the context of TSRDs. Examples included the Clinician-Administered PTSD Scales (CAPS) such as the CAPS-IV-TR^47^ or the CAPS-5,^48^ PTSD Checklist (PCL), RSA, HRV, electrodermal activity (EDA), cardiac pre-ejection period and immunological markers.

Studies were excluded if they (a) did not use VNS as an intervention, (b) reported non-quantitative evaluations of VNS and TRSD-related outcomes, (c) included a non-adult sample or (d) lacked clear indication of the presence of clinical diagnoses of TSRDs.

### Data extraction

Two reviewers (S.G. and T.B.) completed data extraction on 30 March 2023. Study authors were further contacted if there were missing data. Key variables extracted from each study included the following: authors, publication date, geographic region, trial type, primary diagnosis, average age, biological sex proportions, study setting, exposure type, VNS modality, type of comparison group, number of participants, attrition and exclusion rates, length of follow-up, treatment outcomes, sample size, mean scores, standard deviations, event or response rates, reported slopes and frequencies associated with adverse effects.

### Risk of bias, quality assessments and the Grading of Recommendations Assessment, Development and Evaluation framework

Quality assessments were conducted using Cochrane’s risk-of-bias tool (RoB-2) for randomised trials.^49^ Two authors (S.E.A. and R.A.H.) independently conducted risk of bias assessments. Discrepancies not resolved between both authors were further evaluated for resolution by a third author (T.B.). We used the Grading of Recommendations Assessment, Development and Evaluation (GRADE) framework to assess the overall credibility of evidence for each outcome.^50^ The GRADE approach uses several domains to categorise levels of certainty as very low, low, moderate and high. Evidence from randomised controlled trials is initially graded as high, but can then be downgraded to lower levels depending on study limitations (risk of bias), inconsistency, indirectness, imprecision and publication bias. One reviewer (S.K.) graded the overall quality of evidence with the GRADE framework, with decisions checked by a second reviewer (T.B.).

### Data analysis

The state of the evidence on tcVNS included heterogeneous outcomes, overlapping samples and differences in reported effects. As such, a narrative approach was utilised for the synthesis of data. Narrative synthesis clustered findings by outcome type. This included respective reporting of the psychiatric symptoms and psychological experiences, ANS and immunological effects associated with tcVNS comparisons against control conditions in which a non-functional VNS device was used (i.e. sham VNS). Although heterogeneity across studies precluded formal meta-analyses, we conducted a directional analysis by tallying the number of studies reporting positive, negative or null effects for each outcome measure, following the SWiM guidelines.^45^

## Results

**Fig. 1:**
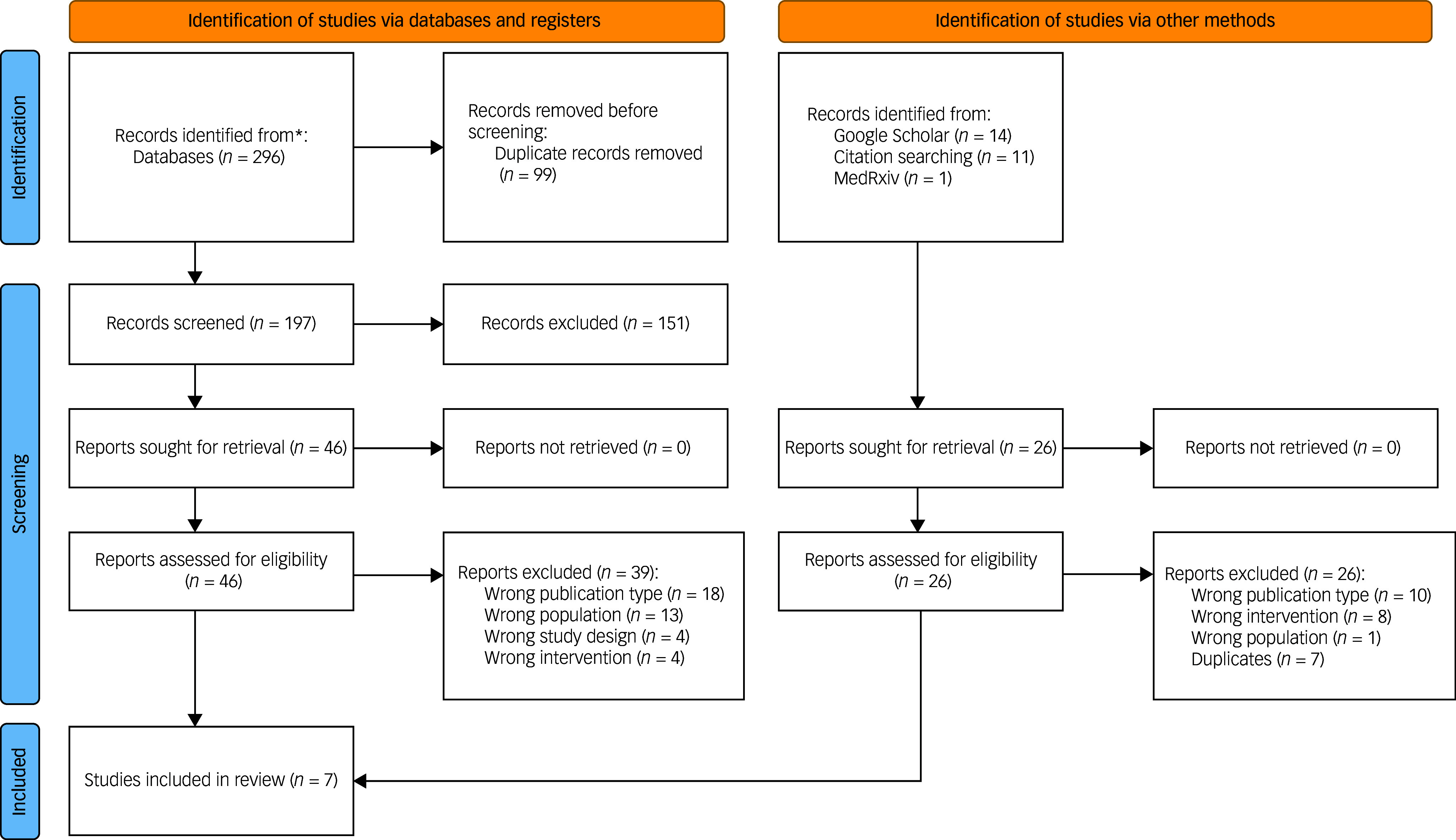
PRISMA flow diagram.^53^ *Includes records identified through Medline, Embase, PsycINFO, CINAHL, Web of Science, the Cochrane Central Register of Controlled Trials (CENTRAL), ISRCTN and ClinicalTrials.gov.

**Fig. 2:**
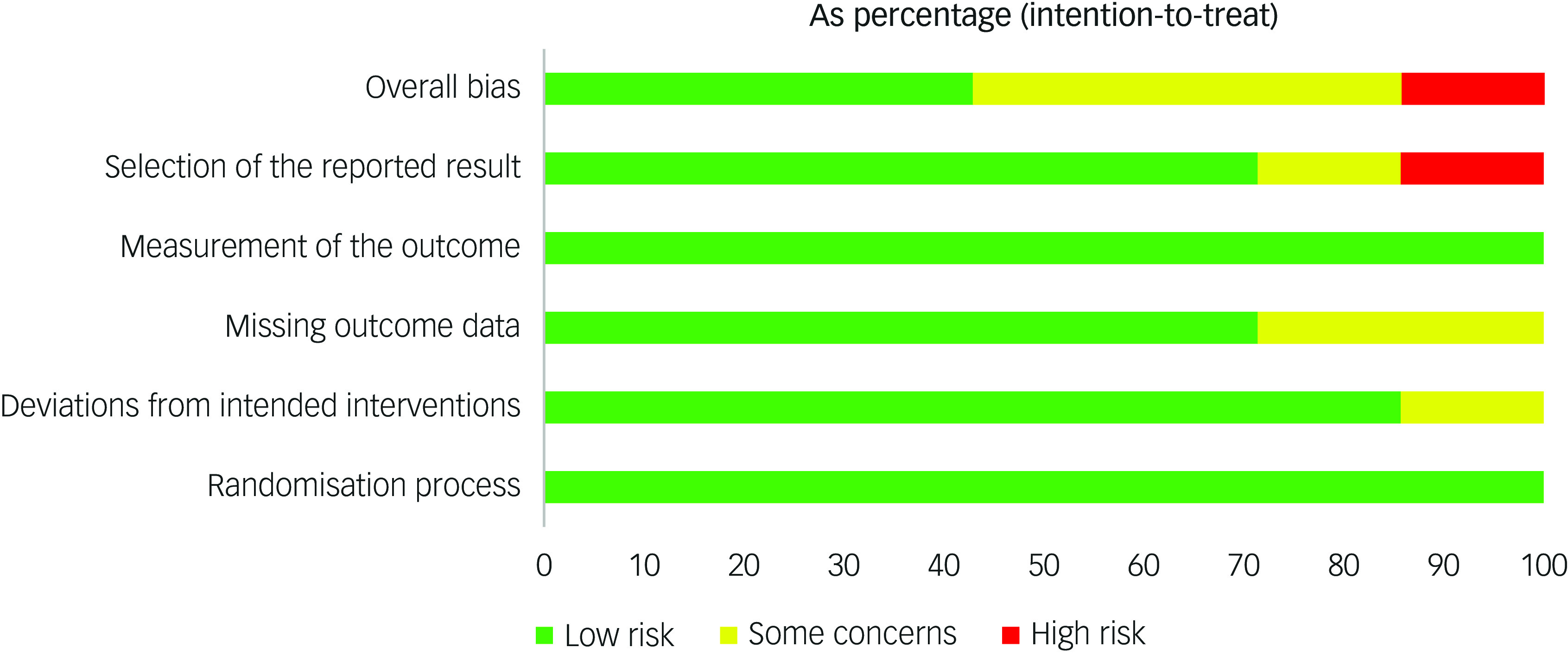
Risk of bias assessment of included studies.

A total of 296 abstracts were identified during our database search of the literature. A supplementary 26 records were identified through other means, such as manual reference searching. Forty-six were selected for full-text screening against study criteria (see Fig. 1). Seven published studies^9,51–52^ met the inclusion criteria for this systematic review (see Table 1). Six of these publications were all sibling studies derived from data collected within the same randomised control trial protocol (identifier NCT02992899). All studies focused on PTSD with no reported outcomes relating to other forms of TSRDs.^9,51–52^ Risk-of-bias analysis showed low or moderate risk. One study deemed as having a highly probable risk of bias owing to alterations of their analysis plan as a result of low statistical power. The overall risk of bias and the associated subdomain’s for included studies are presented in Fig. 2. Pooling of data was not possible across studies because of methodological differences and heterogeneity of outcomes. As such, findings were presented in narrative form.

**Table 1:** Publication data from the registered randomised controlled trial

Study	Study duration (days)	Average age	Comparator	Sample size	Treatment allocation	Outcome	Measures used	TSRD-related findings
Lamb et al, 2017^9^	1	PTSD treatment: mean 30.4 (s.d. = 5.4) Healthy controls: mean 29.7 (s.d. = 7.0)	Active versus sham VNS	*n* = 22	PTSD and TBI group 12 Healthy controls 10	Autonomic nervous system activity	Continuous blood pressure and electrocardiogram	investigated RSA and EDA in a startle-blink paradigm and HRV across different postural tilts, but merged PTSD and healthy controls for analysis, finding an increase in parasympathetic activity with tcVNS in the mixed group. Although specific conclusions about PTSD could not be drawn, RSA variations were noted in PTSD and TBI patients at 30- and 60-degree tilts, suggesting posture-influenced autonomic responses
Bremner et al, 2021^54^^a^	90	PTSD treatment: mean 37 (s.d. = 13) PTSD control: mean 40 (s.d. = 14)	Active versus sham VNS	*n* = 20	VNS 9 Sham VNS 11	Psychiatric symptoms, inflammatory factors, retention rates	CAPS, PCL-Civilian, HRSA, HRSD, CGI, biomarker assay	Active tcVNS enhanced treatment retention (89%) compared with sham VNS (73%). PTSD symptoms were significantly reduced in tcVNS-exposed patients when compared with sham VNS. However, no such difference was observed with CAPS assessments. IL-6 levels were increased following trauma script exposure, and this increase was blunted only in the active sample
Bremner et al, 2020^51^^a^	3	Overall: mean 31 (s.d. = 9) PTSD treatment: mean 29 (s.d. = 8) PTSD controls: mean 32 (s.d. = 8) Healthy treatment: mean 30 (s.d. = 9) Healthy controls: mean 34 (s.d. = 12)	Active versus sham VNS	*n* = 30	PTSD group: VNS 5 Sham VNS 5 Healthy controls: VNS 11 Sham VNS 9	Inflammatory factors, anger	Biomarker assay, visual analogue scales	Active tcVNS significantly reduced stress-induced IL-6 and IFN-γ response post exposure to trauma script and blunted the subjective anger response in the PTSD group compared with sham VNS. No difference in IL-6 response and no mention of differing anger response was noted when comparing healthy active and sham VNS conditions
Wittbrodt et al, 2021^52^^a^	1	PTSD treatment: mean 35.3 (s.d. = 12.7) PTSD control: mean 37.6 (s.d. = 13.7)	Active versus sham VNS	*n* = 22	VNS 11 Sham VNS 11	Psychological distress, anger, anxiousness, fear, nervousness, dissociative symptoms, neuroimaging	High-resolution positron emission tomography scanning with radiolabeled water, subjective units of distress, Clinician-Administered Dissociative States Scale, State-Trait Anger Scale, visual analogue scales	No significant differences in psychological outcomes were found between tcVNS and sham VNS conditions. Neuroimaging, however, revealed distinct patterns: TcVNS selectively activates the anterior cingulate and hippocampus, key for emotional and memory processing, unlike the sham VNS stimulation, which broadly activates frontal, thalamic and insular regions
Gurel et al, 2020^56^^a^	3	Overall: mean 35 (s.d. = 13) PTSD treatment: mean 33 (s.d. = 12) PTSD control: mean 38 (s.d. = 13)	Active versus sham VNS	*n* = 25	VNS 13 Sham VNS 12	Autonomic nervous system activity	Electrocardiography, seismocardiography, photoplethysmography, electrodermal activity, cuff-based blood pressure, respiratory rate	Participants with PTSD were exposed to personalised traumatic scripts and mental stress tasks (public speech and mental arithmetic). This method allowed for a controlled assessment of tcVNS’s effect on autonomic responses to stress. The active tcVNS group showed a decrease in sympathetic arousal compared with the sham VNS group, evidenced by changes in heart rate, photoplethysmogram amplitude and pulse arrival time, indicating a dampening effect on stress-induced autonomic arousal
Gazi et al, 2022^55^^a^	1	Overall: mean 34 (s.d. = 11)	Active versus sham VNS	*n* = 46	PTSD group 24 Healthy controls 26	Autonomic nervous system activity	Respiratory effort (RSP), electrocardiogram, high-resolution positronemission tomography	tcVNS increased heart rate variability measures, such as RMSSD and pNN50, specifically in patients with PTSD, with pNN50 differences notable only in the initial minute and RMSSD throughout the stimulation. Contrarily, in healthy individuals, tcVNS enhanced expiratory time rather than HRV, highlighting that tcVNS effects on autonomic function, including respiratory and heart rate variability, are modulated by PTSD presence
Gazi et al, 2021^53^^a^	1	Overall: mean 36 (s.d. = 12)	Active versus sham VNS	*n* = 24	VNS 13 Sham VNS 11	Autonomic nervous system activity	Electrocardiogram, photoplethysmography, seismocardiography	tcVNS significantly reduced the reactivity of 1/PTT responses to traumatic stress exposure in patients with PTSD compared with sham VNS stimulation, indicating a decrease in stress-induced sympathetic arousal. This effect was specifically observed during traumatic recall, highlighting tcVNS’s potential for reducing sympathetic hyperarousal

a. Indicates sibling study.

PTSD, post-traumatic stress disorder; VNS, vagus nerve stimulation; TBI, traumatic brain injury; RSA, respiratory sinus arrhythmia; EDA, electrodermal activity; HRV, heart rate variability; tcVNS, transcutaneous cervical vagus nerve stimulation; CAPS, Clinician-Administered PTSD Scale; PCL-Civilian, Post-traumatic Stress Disorder Checklist – Civilian version; HRSA, Hamilton Rating Scale for Anxiety; HRSD, Hamilton Rating Scale for Depression; CGI, Clinical Global Impression; IL-6, interleukin-6; IFN-γ, interferon-gamma; RMSSD, root mean square of successive differences between adjacent normal (NN) heart-beat intervals; pNN50, percentage of successive NN intervals that differ by more than 50 ms; RSP, respiratory effort signal; 1/PTT, inverse of pulse transit time.

The sample sizes within the included studies ranged from 20^54^ to 46,^55^ and the average patient age varied from 29 (s.d. = 8)^51^ to 40 (s.d. = 14) years.^54^ All studies were conducted in the USA and included 137 patients with a formal PTSD diagnosis. Notably, all designs identified within the literature followed a randomised controlled trial methodology with double-blinding, and four reported being compliant with the CONSORT statement for transparent reporting of randomised controlled trials.^51,52,54,56^ In all studies, tcVNS was compared with sham VNS treatment. One study provided information on clinically relevant measures of PTSD outcomes and their association with tcVNS exposure.^54^ All authors were further contacted to confirm that they did not have any unpublished data relating to clinical measures of PTSD, and correspondence suggested that our identified study^54^ represents the only randomised controlled trial with clinical assessment of PTSD. Two studies reported psychological outcomes.^52,54^ Outcomes relating to the ANS were included in four studies,^9,53–56^ and two studies provided data relating to immunological markers.^51,54^ Out of the seven included randomised controlled trials, six used repeated tcVNS exposure methodology and trauma scripts for stress induction.^51–52^

### Psychiatric symptoms and central nervous system effects

A single study assessed differences in PTSD symptoms experienced because of tcVNS or sham VNS conditions.^54^ Participants were exposed to personalised trauma scripts, derived from their own traumatic experiences, or neutral scripts with emotionally non-arousing content. This dual-script exposure design was instrumental in isolating the specific responses elicited by trauma-relevant versus neutral stimuli. CAPS-5 and PCL measurements demonstrated a decrease in PTSD symptom across the entire sample. However, the results of comparison between active tcVNS and sham group were dependent on the assessment method. A significant decrease of 31% was observed in the tcVNS group when compared with the sham VNS condition, when assessing self-reported PCL reports of PTSD severity. This pattern was further maintained when examining the hyperarousal subscale of the PCL scale. However, no significant difference between the active and sham VNS group was present when comparing outcomes as measured by the CAPS-5. The tcVNS group was further found to experience less somatic anxiety symptoms, as measured by the Hamilton Rating Scale for Anxiety,^57^ when compared with sham VNS exposure. Notably, tcVNS did not significantly differ from sham VNS conditions when evaluating depressive symptoms, as measured by the Hamilton Rating Scale for Depression.^58^ In this study, the use of tcVNS was well-tolerated by participants, with a 16% improvement in treatment drop-out rate in patients with PTSD receiving tcVNS when compared with sham VNS conditions. Following their trial, open-label administration of tcVNS was reported to produce improvement in overall psychiatric symptoms at124 days following study initiation, suggesting a positive therapeutic response to tcVNS in patients with PTSD. In an earlier trial, the same authors observed a reduction in anger responses in patients with PTSD when compared with participants without PTSD during exposure to a personal trauma script, as measured by a visual analogue scale ranging from 0 to 100.^51^ Within the PTSD sample, patients receiving active tcVNS treatment rather than sham VNS stimulation were further found to be associated with a decrease in anger experiences when exposed to a personalised exposure script. No such difference was reported when comparing active and sham VNS conditions within healthy members of their sample.

Another randomised controlled trial provided information on phenomenological differences in distress experienced during exposure to trauma and neutral scripts.^52^ To obtain their sample, trained staff conducted a structured interview following the Structured Clinical Interview for DSM-5 disorders (SCID-5)^59^ and used the CAPS-5 to determine the presence of PTSD, which was required for inclusion in their study. No difference between active and sham VNS conditions was established in anger, anxiousness, fear and nervousness as determined by the visual analogue ratings of participants. Moreover, no differences in dissociation were observed between the two conditions. Similarly, there was no significant difference in reported psychological distress within their sample when comparing tcVNS and sham VNS conditions across exposure conditions. Despite the lack of difference in these psychological measures, differences in brain imaging as an outcome of receiving tcVNS were identified. Most notable, tcVNS was found to produce greater activity in the anterior cingulate cortex (ACC) and its subdivisions, such as the subgenual, anterior dorsal and posterior dorsal regions. In contrast, sham VNS exposure was associated with decreased ACC activity. In addition to increased insula activity, sham VNS exposure was found to predict greater left frontal, precentral gyrus and thalamus activity when compared with active tcVNS. Moreover, increased hippocampal activity was only observed to be associated with tcVNS exposure. Overall, findings suggest that tcVNS encourages central nervous system changes that, within the context of PTSD, have been associated with improved autonomic control and emotion regulation capacity. Table 2 summarises the results. It was not possible to calculate a common effect size because of the range of outcomes measured.

**Table 2:** Summary table of results

Outcomes	Number of studies	
	Difference	No difference
Psychological symptoms		
Post-traumatic stress^a^	1	0
Depression	0	1
Anxiety	0	1
Subjective distress (Structured Clinical Interview for DSM-5 disorders)	0	1
Anger	1	0
Autonomic and central nervous system^b^		
Trauma	4	0
Neutral script exposure	2	1

a. On some measures.

b. One study contained healthy participants as well as people with post-traumatic stress disorder.

### Autonomic nervous system

Gurel and colleagues^56^ reported greater parasympathetic activity changes in participants within the tcVNS group compared with sham VNS conditions. Their sample was entirely composed of participants with PTSD, confirmed by SCID and CAPS assessments. Notably, these changes towards parasympathetic dominance were based on tcVNS having significant effects on non-linear HRV (i.e. Poincare-based short-term HRV/Poincare-based short- and long-term HRV; SD1/SD2), photoplethysmogram amplitude and pulse arrival time (PAT) measures. These Poincare-based SD1/SD2 ratios capture short- and long-term fluctuations in heart rhythm and enable distinctions between PNS and SNS inputs, complementing the observed increases in photoplethysmogram amplitude and decreases in PAT that collectively point towards enhanced parasympathetic drive. The increased SD1/SD2 ratio is reflective of more beat-to-beat variability governed by parasympathetic influences, higher photoplethysmogram amplitude indicates parasympathetic-mediated vasodilation and shorter PAT suggests increased parasympathetic modulation of heart rate, collectively demonstrating a shift towards parasympathetic dominance. Concurrently, a decrease in heart rate was further observed. These observations were made within neutral exposure conditions, in which no differences in frequency-based HRV, EDA, respiratory factors, seismocardiography and blood pressure measurements were reported. Findings during traumatic script exposure conditions converged with the aforementioned neutral exposure results. During stress-inducing tasks (e.g. public speaking), participants exposed to tcVNS displayed an increased pre-ejection period (i.e. SNS withdrawal), indicating decreased sympathetic arousal and decreased vascular constriction as measured by pulse pressure, which may reflect an increase PNS activity or convergence towards a decrease in SNS influence. In this context, a lengthening of the pre-ejection period (i.e. the interval from ventricular depolarisation to aortic valve opening) further underscores reduced sympathetic outflow.

Gazi and colleagues expanded upon the work conducted by Gurel et al,^56^ examining parasympathetic activity by using the reciprocal of pulse transit time (1/PTT) because of its greater robustness and decreased influence from variability in assessments of pre-ejection periods.^53^ Within their sample of participants diagnosed with PTSD, percentage differences in 1/PTT were significantly different between tcVNS and sham VNS conditions, but only when patients were exposed to a traumatic script. This difference was present during and within the first minute of exposure. Examinations of neutral stimulus exposure and after the first-minute assessments did not differ between active and control conditions. A further study by the same authors reported an increased level of root-mean-square of successive differences (RMSSD) of normal-to-normal intervals and percentage of successive normal-to-normal intervals that differ by more than 50 ms (pNN50) in the tcVNS group when compared with sham VNS conditions when looking at their sample with PTSD.^55^ Because RMSSD and pNN50 assess short-term beat-to-beat variability, they serve as sensitive indices of vagal tone, consistent with the observed increases under tcVNS. Differences in pNN50 were only observed as present during the first minute of tcVNS, whereas greater RMSSD activity was present during the entire 2min of vagal stimulation. Notably, tcVNS-induced differences from sham VNS were observed to depend on the presence of PTSD. Within their healthy sample, an increase in expiratory time was noted in the tcVNS group post-stimulation when compared with sham VNS. In contrast, HRV rather than respiratory rate was observed to differ during the 2 min of tcVNS stimulation as opposed to post stimulation when compared with sham VNS and active PTSD groups.

A final study evaluated RSA and EDA activity within the context of a startle-blink paradigm and examined HRV at different postural tilting angles.^9^ However, because of sampling limitations, they pooled their healthy controls with their PTSD group during quantitative analyses. Within their mixed sample (i.e. inclusive of non-PTSD participants), an 0.88 standard deviation increase in parasympathetic activity attributable to tcVNS was detected. RSA, which reflects vagal influence on heart rate across the respiratory cycle, and EDA, a measure of sympathetic-driven skin conductance, both supported this increase in parasympathetic tone. Although these results do not allow extrapolation specific to PTSD, visual inspection of their provided figure of patients living with PTSD and traumatic brain injury displays RSA differences during measurements at 30- and 60-degree postural tilting angles. Table 2 summarises the results. As before, it was not possible to calculate a common effect size because of the range of outcomes measured.

### Inflammatory response to VNS

Within their 2021 publication, Bremner and colleagues reported the presence of an increased interleukin-6 concentration in participants within the sham VNS condition when compared with tcVNS conditions. However, it is pivotal to note that this observation was present both before and after administration of tcVNS.^54^ In contrast, within their 2020 trial, Bremner and colleagues identified an increase in interleukin-6 and interferon-γ following exposure to a personalised trauma script. This inflammatory difference was further demonstrated to be greater in patients with PTSD when compared with the immune response of healthy controls. Notably, tcVNS was determined to block the expected increased levels of interleukin-6 and interferon-γ when compared with sham VNS conditions.^51^ No differences were observed in interleukin-2, interleukin-1β and tumour necrosis factor-α.

### GRADE assessments

Table 3 presents the GRADE assessments for the eight outcomes of interest. Entries for risk of bias and inconsistency were derived from Fig. 2. In terms of indirectness, all studies were rated as having serious limitations largely because of concerns about whether the population and/or intervention differed from those that might be relevant for the wider population. All outcomes were rated as having severe limitations under imprecision, because data came from one or two small studies.

**Table 3:** GRADE ratings for evidence certainty

Outcomes	Quality assessment					
	Risk of bias	Inconsistency	Indirectness	Imprecision	Publication bias	Certainty
Post-traumatic stress	Moderate	Not applicable	Serious limitations	Serious limitations	Moderate	Very low
Depression	Moderate	Not applicable	Serious limitations	Serious limitations	Moderate	Very low
Anxiety	Moderate	Not applicable	Serious limitations	Serious limitations	Moderate	Very low
Distress	Moderate	Not applicable	Serious limitations	Serious limitations	Moderate	Very low
Anger	Moderate	Not applicable	Serious limitations	No serious limitations	Moderate	Very low
Autonomic and central nervous system – trauma	Moderate	Not applicable	Serious limitations	Serious limitations	Moderate	Very low
Autonomic and central nervous system – neutral script exposure	Moderate	Not applicable	Serious limitations	Serious limitations	Moderate	Very low
Inflammatory response	Moderate	Not applicable	Serious limitations	Serious limitations	Moderate	Very low

Given the impossibility to test for publication bias by using tests of funnel asymmetry, this was assessed in terms of the number and size of studies, or possible conflicts of interest in study sponsors. Despite there being relatively few unpublished studies, all outcomes were rated as being at moderate risk of publication bias because of the limited number of small studies. Notably, five out of the seven studies^51,54,55–52^ reported either having an author that received funding from a device manufacturer or having obtained the devices for their study by the manufacturer free of charge. However, no evidence was available to assess whether these disclosures influenced the findings reported in the studies.

## Discussion

To our knowledge, the present systematic review represents the first evidence synthesis on tcVNS interventions for TSRDs. However, all papers identified were focused on PTSD, demonstrating a lack of any identifiable data point describing tcVNS use in non-PTSD forms of TSRDs. Although we searched databases from inception to present, publication dates ranged from 2017 and 2022, confirming the novelty of VNS research within the field of biological psychology and psychiatry. Although all seven identified papers used a robust methodology in which randomisation and blinding were present, only one study was interventional in a clinically relevant way reporting PTSD symptom outcomes. Therefore, the overall quality of evidence for the treatment of PTSD with tcVNS is very low. Moreover, the limited number of small studies and moderate overall risk of bias meant that the certainty of the overall evidence was also very low. Moreover, six out of the seven publications on the topic are all derived from the same data collection effort. Thus, findings are not yet replicated enough to entail applicability to the patient population. In addition, despite our comprehensive database search without geographical restrictions, all identified studies were conducted in the USA, limiting our understanding of tcVNS efficacy across different healthcare systems and cultural contexts. This geographical homogeneity is particularly significant, given that PTSD manifestation and treatment responses can vary substantially across cultural settings^60^ and healthcare delivery approaches.^61^ The generalisability of current findings to diverse global populations therefore remains uncertain and in need of multicentre international trials.

Currently, first-line treatment for PTSD involves psychotherapy-based care, such as cognitive–behavioural therapy^62,63^ and eye movement desensitisation and reprocessing,^64^ and antidepressant medication, including selective serotonin reuptake inhibitors and serotonin and norepinephrine reuptake inhibitors.^65^ Despite the availability of effective interventions, not all patients experience recovery.^66^ We therefore conducted this systematic review to determine the effect of tcVNS in producing sustained change in PTSD outcomes and, more broadly, TSRDs. Contrary to anticipation, the present state of evidence on tcVNS as a treatment intervention for individuals with PTSD was limited and not sufficient for clinical practice, as demonstrated by evidence synthesis. Although all studies focused on clinical samples living with PTSD, evidence of psychiatric symptom response was only present in one randomised controlled trial evaluation.^54^ Moreover, differences in PTSD symptom experience within the study depended on the symptom measurement type, with non-significant results associated with using CAPS and significant changes only observed in self-reported measurements of PTSD severity (i.e. PCL). Furthermore, no information relating to remission outcomes or the number needed to treat was reported within the literature, suggesting that available evidence relating to VNS for the treatment of PTSD is not currently considering critical clinical measures of treatment efficacy.

Most of the literature examined physiological responses, and most examined the effect of VNS within both neutral and stress exposure conditions. This review provides tentative evidence of autonomic modulation caused by VNS treatment. Notably, evidence synthesis on patients with PTSD reflects similar physiological responses to reports of VNS use in healthy controls.^67^ Our systematic review uncovered preliminary evidence demonstrating differences in cerebral activity attributable to direct VNS. Notably, tcVNS induced marked increases in activity within the ACC, including its subgenual, anterior dorsal and posterior dorsal subdivisions, and the left hippocampus, areas critically involved in the processing of emotions^68,69^ and memory.^70^ These regions have been consistently implicated in the pathophysiology of PTSD, serving as key neural substrates for the disorder’s characteristic symptoms of dysregulated affect and intrusive memories.^71,72^ In contrast, sham VNS stimulation failed to specifically target the neural pathways associated with therapeutic benefits observed in the tcVNS group. Thus, our finding lends some support to the NVIT as it suggests the presence of direct influence exerted by vagal nerve alterations and neurocognitive processes. Additionally, observed differences in hippocampal activity may reflect the strong influence associated with tcVNS treatments. Although this was only examined by one randomised controlled trial within the literature, previous evidence has demonstrated decreased hippocampal volumes within individuals with PTSD^73^ and a diminished volume that plays a role in PTSD-related processes such as avoidance-based and extinction learning, avoidance symptoms,^74,75^ emotional memory formation, consolidation and retrieval.^76^ Considering the presence of dysregulation in HPA negative feedback within PTSD and the role of the hippocampus in both modulating and attenuating HPA activity during chronic stress experiences,^77^ current evidence warrants further replication and extension examining temporal precedence in which tcVNS-induced alterations of hippocampal activity may further promote the enhanced HPA control. In the broader literature, evidence of marked systemic inflammation in patients with PTSD is robust, and its outcomes are related to multisystemic illness development, such as in the case of cardiovascular and digestive diseases.^78^ Within our synthesis, evidence on pro-inflammatory response was non-significant. However, literature pertaining to IL-6 expression provided mixed findings. As such, future examination of specific subtypes of immune messengers is of great value.

The current evidence provides preliminary experimental validation that serves as an initial proof of concept for the premise that VNS may alter specific aspects associated with PTSD phenomenology, rather than the totality of PTSD-related morbidity. This is reflected in the overall certainty of evidence being rated as very low, using the GRADE framework. Future studies should evaluate tcVNS as an adjunct to first-line interventions. This approach would enable assessment of both multimodal efficacy and treatment response times. Particular attention should focus on how tcVNS may facilitate the resolution of specific symptoms, notably hyperarousal, during standard therapeutic protocols. Furthermore, the current evidence suggests that timing may be necessary when considering this therapeutic modality. For instance, Gazi et al^53^ reported acute effects within 2 min of exposure, and evidence suggests tcVNS as potentially reducing symptomatic experience during stress induction,^56^ rather than providing overall relief of PTSD-related experiences. Future evidence should consider increasing methodological homogeneity to the extent that meta-analysis becomes possible; with the inclusion of PTSD-related symptom experience, the evidence generated could provide greater clarity on the role of tcVNS as a potential intervention for clinicians. Notably, most evaluations of autonomic activity resulting from tcVNS application were transient. There remains a need to explore the long-term effects of tcVNS on the ANS, particularly by comparing outcomes of repeated booster administrations with those of single-day usage. We also recommend that studies examining tcVNS within the context of psychiatric illness emphasise symptom outcome-related data collection and analysis, even if done as a secondary outcome; thus generating much-needed evidence on its efficacy for further meta-analytical evaluation of its potential benefits to patient populations. Similarly, considering the state of evidence and the portability of tcVNS devices, clinical trials should evaluate its potential in acute event management. Overall, data on symptomatic outcomes during exposure to stress inductive stimuli remains dire. Upon thorough examination of the evidence, which is limited to findings from two small-scale studies, it becomes apparent that the contemporary clinical scientific literature is devoid of sufficient empirical support for the theoretical claims suggesting that tcVNS exerts a direct effect on the symptoms of PTSD. This lack of evidence shows that we cannot promote these theories as clinically relevant for treatment at this time.

### Clinical implications

From the vantage point of practising psychiatrists and psychotherapists, these findings underscore both the promise and the practical hurdles of adopting tcVNS. Although standard treatments such as psychotherapy and appropriate pharmacotherapies remain the bedrock of PTSD care, emerging evidence hints that tcVNS might help stabilise hyperarousal symptoms during therapy sessions. In particular, its physiological target may open avenues for patients who have demonstrated limited response to conventional strategies, offering an adjunctive option that could potentially address residual stress reactivity and autonomic dysregulation. However, without robust clinical trials and more consistent methodological approaches, integrating tcVNS into routine practice would be premature. The conceptual alignment of tcVNS with established psychotherapeutic frameworks nonetheless warrants further exploration, as combining neuromodulatory techniques with evidence-based psychotherapy could yield synergistic effects, thereby refining and personalising PTSD interventions.

In conclusion, theories relating to autonomic response may provide a framework for future research on tcVNS and TSRD-related outcomes. Currently, there remains no empirical evaluations of tcVNS within the context of treating non-PTSD forms of TSRDs. Notably, the current literature assessing the role of vagal stimulation in treating PTSD does not meet evidence-based medicine standards, and clinicians should remain wary of its utility in treating patients with TSRDs. Although preliminary evidence does provide an early-stage proof of concept for the induction of PNS dominance as a result of tcVNS exposure, the effects of tcVNS seem to be short-lasting, and the effect of repeated administration on longitudinal outcomes within the ANS remains unknown. The preliminary evidence of autonomic modulation through tcVNS, particularly its effects on hyperarousal and anger responses, represents a potentially novel pathway for therapeutic intervention in trauma-related disorders. However, substantial empirical validation through rigorous clinical trials specifically assessing PTSD prognosis remain essential before any therapeutic applications can be considered.

## Supporting information

Benzouak et al. supplementary material Benzouak et al. supplementary material

## Supplementary material

The supplementary material is available online at https://doi.org/10.1192/bjo.2025.10057
