# Supplementary material for: Transcutaneous vagal nerve stimulation for the treatment of trauma- and stressor-related disorders: systematic review of randomised controlled studies
Source: BJPsych Open. 2025 Aug 1;11(5):e165. doi: 10.1192/bjo.2025.10057 (PMC12344430; doi:10.1192/bjo.2025.10057)
Supplement: Benzouak et al. supplementary material [file S2056472425100574sup001.docx]

**S-1: Search Strategy**

**OVID: MEDLINE, EMBASE, PSYCHINFO, COCHRANE CENTRAL**

1. vagus nerve stimulation/

2. ((Vagal adj2 stimulation) or (Vagus adj2 stimulation) or VNS).ti,ab.

3. 1 or 2

4. posttraumatic stress disorder/

5. acute stress disorder/

6. reactive attachment disorder/

7. adjustment disorder/

8. ((Disinhibited adj1 Social adj1 Engagement adj1 Disorder) or (Stress adj5 disorder) or (Trauma adj5 disorder) or (reactive adj1 attachment adj disorder) or (adjustment adj1 disorder)).ti,ab.

9. 4 or 5 or 6 or 7 or 8

10. 3 and 9

**CINAHL**

1. (MH "vagus nerve stimulation") OR (((TI Vagal OR AB Vagal) N2 (TI stimulation OR AB stimulation)) OR ((TI Vagus OR AB Vagus) N2 (TI stimulation OR AB stimulation)) OR (TI VNS OR AB VNS))

AND

2. (MH "posttraumatic stress disorder") OR (MH "acute stress disorder") OR (MH "reactive attachment disorder") OR (MH "adjustment disorder") OR (((TI Disinhibited OR AB Disinhibited) N1 (TI Social OR AB Social) N1 (TI Engagement OR AB Engagement) N1 (TI Disorder OR AB Disorder)) OR ((TI Stress OR AB Stress) N5 (TI disorder OR AB disorder)) OR ((TI Trauma OR AB Trauma) N5 (TI disorder OR AB disorder)) OR ((TI reactive OR AB reactive) N1 (TI attachment OR AB attachment) W1 (TI disorder OR AB disorder)) OR ((TI adjustment OR AB adjustment) N1 (TI disorder OR AB disorder)))

**Web of Science**

1. ALL="vagus nerve stimulation"

2. (TI=((Vagal NEAR/2 stimulation ) OR (Vagus NEAR/2 stimulation ) OR VNS ) OR AB=((Vagal NEAR/2 stimulation ) OR (Vagus NEAR/2 stimulation ) OR VNS ))

3. #1 OR #2

4. ALL="posttraumatic stress disorder"

5. ALL="acute stress disorder"

6. ALL="reactive attachment disorder"

7. ALL="adjustment disorder"

8. (TI=((Disinhibited NEAR/1 Social NEAR/1 Engagement NEAR/1 Disorder ) OR (Stress NEAR/5 disorder ) OR (Trauma NEAR/5 disorder ) OR (reactive NEAR/1 attachement NEAR/0 disorder ) OR (adjustement NEAR/1 disorder )) OR AB=((Disinhibited NEAR/1 Social NEAR/1 Engagement NEAR/1 Disorder ) OR (Stress NEAR/5 disorder ) OR (Trauma NEAR/5 disorder ) OR (reactive NEAR/1 attachement NEAR/0 disorder ) OR (adjustement NEAR/1 disorder )))

9. #4 OR #5 OR #6 OR #7 OR #8

10. #3 AND #9
